# Stem cell-based therapy in periodontal regeneration: a systematic review and meta-analysis of clinical studies

**DOI:** 10.1186/s12903-023-03186-6

**Published:** 2023-07-15

**Authors:** Liang Sun, Xinya Du, Huifang Kuang, Honglan Sun, Wen Luo, Chao Yang

**Affiliations:** 1 Center of Stomatology, The Second Affiliated Hospital of Soochow University, No 1055 Sanxiang Road, 215004 Soochow, Jiangsu China; 2 Department of Stomatology, The People’s Hospital of Longhua, 38 Jinglong Jianshe Road, 518109 Shenzhen, Guangdong China; 3 Department of Stomatology, The First Affiliated Hospital of Hainan Medical University, 570102 Haikou, Hainan, China; 4 School of Stomatology, Hainan Medical University, 571199 Haikou, Hainan, China; 5 Research and Development Department, Shenzhen Uni-medica technology Co., Ltd, Liuxian Culture Park, 518051 Shenzhen, Guangdong China

**Keywords:** Mesenchymal stem cells, Meta-analysis, Periodontal regeneration, Stem cell-based therapy, Clinical studies

## Abstract

**Background:**

Periodontitis is a common and chronic inflammatory disease characterized by irreversible destruction of the tooth surrounding tissues, especially intrabony defects, which eventually lead to tooth loss. In recent years, stem cell-based therapy for periodontitis has been gradually applied to the clinic, but whether stem cell-based therapy plays a positive role in periodontal regeneration is unclear at present.

**Methods:**

The clinical studies related to the evaluation of mesenchymal stem cells for periodontal regeneration in PubMed, Cochrane Central Register of Controlled trials (CENTRAL), Web of Science (WOS), Embase, Scopus, Wanfang and China national knowledge infrastructure (CNKI) databases were searched in June 2023. The inclusion criteria required the studies to compare the efficacy of stem cell-based therapy with stem cell free therapy for the treatment periodontitis, and to have a follow-up for at least six months. Two evaluators searched, screened, and assessed the quality and the risk of bias in the included studies independently. Review Manager 5.4 software was used to perform the meta-analysis, and GRADEpro GDT was used to evaluate the level of the evidence.

**Results:**

Five randomized controlled trials (RCTs) including 118 patients were analyzed. The results of this meta-analysis demonstrated that stem cell-based therapy showed better therapeutic effects on clinical attachment level (CAL) (MD = − 1.18, 95% CI = − 1.55, − 0.80, P < 0.00001), pocket probing depth (PPD) (MD = − 0.75, 95% CI = − 1.35, − 0.14, P = 0.020), and linear distance from bone crest to bottom of defect (BC-BD)( MD = − 0.95, 95% CI = − 1.67, − 0.23, P = 0.010) compared with cell-free group. However, stem cell-based therapy presented insignificant effects on gingival recession (P = 0.14), linear distance from cementoenamel junction to bottom of defect (P = 0.05).

**Conclusion:**

The results demonstrate that stem cell-based therapy may be beneficial for CAL, PPD and BC-BD. Due to the limited number of studies included, the strength of the results in this analysis was affected to a certain extent. The high‐quality RCTs with large sample size, multi-blind, multi-centric are still required, and the methodological and normative clinical study protocol should be established and executed in the future.

## Introduction

The periodontium is a complex functional unit consisting of gingiva, alveolar bone, periodontal ligament and cementum. Periodontal ligament plays a critical structural role in bridging cementum and alveolar bone. This functional unit provides an attachment apparatus for the jaw bone and withstand masticatory forces [1]. Periodontitis as a highly prevalent infectious oral disease of periodontium affects more than 50% of all adults [2]. As the disease continues to advance, periodontitis can lead to progressive and irreversible damage of the periodontium, including gingival recessions, loss of soft tissue attachment and intrabony defect, which eventually results in premature tooth loss [3].

Periodontitis may begin in childhood or later, and usually manifest symptoms in adulthood. As the main and important pathogens, a variety of bacteria play a vital role in the initiation and development of periodontitis [4]. Dental plaque is always forming on the teeth, especially between the teeth and the gingiva. The accumulation of dental plaque will cause the host’s immune response and subsequent inflammation [2]. The goal of conventional periodontal therapy is to prevent the inflammation and the aggressive progression of periodontitis. However, as mentioned above, one of the characters of this disease is irretrievable destruction of the tooth-supporting tissues. Nonsurgical or surgical treatment, including oral hygiene instruction, full-mouth scaling and root planning can control infections and arrest the disease progression, but cannot achieve complete periodontal regeneration defined by The American Academy of Periodontology, as the formation of new cementum, alveolar bone, and functional periodontal ligament on the periodontitis root surface [5–7]. Therefore, there is a need to find new therapeutic strategies to achieve complete periodontal regeneration. In the past few years, it seems that stem cells-based tissue engineering has the potential to be an innovative method to induce functional periodontal tissue regeneration as suggested by some of previous pre-clinical and clinical reports.

Stem cells, scaffold and growth factors are the three basic and key biologic elements of tissue engineering [8]. To date, mesenchymal stem cells (MSCs) are the most commonly used stem cells in tissue engineering, and various MSCs have been isolated from different tissue sources, including the bone marrow, adipose, perinatal tissues, dental tissues and so forth. Up to now, about six types of dental stem cells could be isolated from the different developmental phase of teeth or different age of donor, including stem cells from human exfoliated deciduous teeth (SHED), dental follicle stem cells (DFSCs), stem cells from apical papilla (SCAP), dental pulp stem cells (DPSCs), periodontal ligament stem cells (PDLSCs), and gingival stem cells (GSCs) [9]. For periodontal tissue engineering, dental stem cells, especially autologous dental stem cells are the most commonly used in recent clinical applications [6, 7, 10–12]. One case report also suggested that allogeneic SHEDs of a youth 7-year-old donor were grafted to treat the periodontitis of a 61-year-old patient [13].

Several previous systematic reviews and meta-analysis of preclinical studies with stem cell therapies for periodontal tissue regeneration have been published, and demonstrated that transplantation of MSCs can be considered a promising and beneficial strategy for periodontal regeneration. Moreover, another literature of a systematic review and meta-analysis also evaluated the potential of MSC transplantation on periodontal regeneration for clinical applications. However, only two clinical studies were included in the quantitative analysis of this literature, which may limit the significance of the results. Some new studies about periodontal tissue regeneration with clinical applications of MSCs have been published recently. The goal of this systematic review and meta-analysis is to evaluate the efficacy and safety of stem cell-based therapy in periodontal regeneration via performing estimate of the previous and recent clinical applications.

## Materials and methods

### Study design

This systematic review and meta-analysis were conducted according to the standard guideline of “Preferred Reporting Items for Systematic Reviews and Meta-Analyses (PRISMA)” [14].

### Inclusion criteria of the studies

All the published studies about randomized controlled trial (RCT) of MSCs for periodontal regeneration were considered. No matter blinding or allocation hiding was accepted. The eligibility criteria for the patients were: adults older than 20 years of age, diagnosis of moderate-severe chronic periodontitis or in stages III-IV, clinical attachment level (CAL) ≥ 6 mm, absence of systemic or immunological disease, nonsmoking, non-pregnancy and lactation, no history of periodontal surgery or restoration. After a standard complete full mouth nonsurgical periodontal treatment, including supragingival scaling, subgingival root planning, the patients in test group received MSC-based therapy, and the patients in control group received the same therapeutic schedule only without MSCs. The main outcome of the treatment including CAL, pocket probing depth (PPD), recession of the gingival margin (REC), linear distance from cementoenamel junction to bottom of defect on radiographs (CEJ-BD), linear distance from bone crest to bottom of defect (BC-BD), and other outcomes. Exclusion criteria of the studies were: preclinical studies, article type being review and case report, follow-up period being less than six months, the study not containing complete original data, the clinical study not concerning stem cells.

### Search strategy

An extensive search of the literatures was conducted in June 2023. Eligible studies were retrieved from PubMed, Cochrane Central Register of Controlled trials (CENTRAL), Web of Science (WOS), Embase, Scopus, Wanfang and China national knowledge infrastructure (CNKI) databases. The article type filters as “Clinical Trial” and “Randomized Controlled Trial” were applied for PubMed search, and type filters as “Clinical Trial” was applied for WOS search. The key words for search strategy are as follows: “periodontal regeneration”, “periodontitis”, “stem cell”, “mesenchymal stem cell”, “mesenchymal stromal cell”, “dental stem cell”, “periodontal ligament stem cell”, “dental pulp stem cell”, “gingival mesenchymal stem cells”, “umbilical cord mesenchymal stem cell”, “bone marrow mesenchymal stem cell”, “clinical trial”, “clinical study”. The search strategies in the aforementioned databases are reported in Table 1.

**Table 1:** The details of search strategy in databases

Database	Query	Results
Pubmed	((periodontal regeneration OR periodontitis) AND stem cell) OR ((periodontal regeneration OR periodontitis) AND mesenchymal stem cell) OR ((periodontal regeneration OR periodontitis) AND mesenchymal stromal cell) OR ((periodontal regeneration OR periodontitis) AND dental stem cell) OR ((periodontal regeneration OR periodontitis) AND periodontal ligament stem cell) OR ((periodontal regeneration OR periodontitis) AND dental pulp stem cell) OR ((periodontal regeneration OR periodontitis) AND gingival mesenchymal stem cell) OR ((periodontal regeneration OR periodontitis) AND umbilical cord mesenchymal stem cell) OR ((periodontal regeneration OR periodontitis) AND bone marrow mesenchymal stem cell)	47
CENTRAL	((periodontal regeneration OR periodontitis) AND stem cell) OR ((periodontal regeneration OR periodontitis) AND mesenchymal stem cell) OR ((periodontal regeneration OR periodontitis) AND mesenchymal stromal cell) OR ((periodontal regeneration OR periodontitis) AND dental stem cell) OR ((periodontal regeneration OR periodontal ligament stem cell) AND stem cell) OR ((periodontal regeneration OR periodontitis) AND dental pulp stem cell) OR ((periodontal regeneration OR periodontitis) AND gingival mesenchymal stem cell) OR ((periodontal regeneration OR periodontitis) AND umbilical cord mesenchymal stem cell) OR ((periodontal regeneration OR periodontitis) AND bone marrow mesenchymal stem cell) in All Text	105
WOS	((periodontal regeneration OR periodontitis) AND stem cell) OR ((periodontal regeneration OR periodontitis) AND mesenchymal stem cell) OR ((periodontal regeneration OR periodontitis) AND mesenchymal stromal cell) OR ((periodontal regeneration OR periodontitis) AND dental stem cell) OR ((periodontal regeneration OR periodontitis) AND periodontal ligament stem cell) OR ((periodontal regeneration OR periodontitis) AND dental pulp stem cell) OR ((periodontal regeneration OR periodontitis) AND gingival mesenchymal stem cell) OR ((periodontal regeneration OR periodontitis) AND umbilical cord mesenchymal stem cell) OR ((periodontal regeneration OR periodontitis) AND bone marrow mesenchymal stem cell)	47
Embase	(‘periodontal regeneration’/exp OR ‘periodontal regeneration’ OR (periodontal AND (‘regeneration’/exp OR regeneration)) OR ‘periodontitis’/exp OR periodontitis) AND (‘stem cell’/exp OR ‘stem cell’ OR ((‘stem’/exp OR stem) AND (‘cell’/exp OR cell))) AND (‘clinical’/exp OR clinical) OR ((‘periodontal regeneration’/exp OR ‘periodontal regeneration’ OR (periodontal AND (‘regeneration’/exp OR regeneration)) OR ‘periodontitis’/exp OR periodontitis) AND (‘mesenchymal stem cell’/exp OR ‘mesenchymal stem cell’ OR (mesenchymal AND (‘stem’/exp OR stem) AND (‘cell’/exp OR cell))) AND (‘clinical’/exp OR clinical)) OR ((‘periodontal regeneration’/exp OR ‘periodontal regeneration’ OR (periodontal AND (‘regeneration’/exp OR regeneration)) OR ‘periodontitis’/exp OR periodontitis) AND (‘mesenchymal stromal cell’/exp OR ‘mesenchymal stromal cell’ OR (mesenchymal AND stromal AND (‘cell’/exp OR cell))) AND (‘clinical’/exp OR clinical)) OR ((‘periodontal regeneration’/exp OR ‘periodontal regeneration’ OR (periodontal AND (‘regeneration’/exp OR regeneration)) OR ‘periodontitis’/exp OR periodontitis) AND (‘dental stem cell’/exp OR ‘dental stem cell’ OR ((‘dental’/exp OR dental) AND (‘stem’/exp OR stem) AND (‘cell’/exp OR cell))) AND (‘clinical’/exp OR clinical)) OR ((‘periodontal regeneration’/exp OR ‘periodontal regeneration’ OR (periodontal AND (‘regeneration’/exp OR regeneration)) OR ‘periodontitis’/exp OR periodontitis) AND (‘periodontal ligament stem cell’/exp OR ‘periodontal ligament stem cell’ OR (periodontal AND (‘ligament’/exp OR ligament) AND (‘stem’/exp OR stem) AND (‘cell’/exp OR cell))) AND (‘clinical’/exp OR clinical)) OR ((‘periodontal regeneration’/exp OR ‘periodontal regeneration’ OR (periodontal AND (‘regeneration’/exp OR regeneration)) OR ‘periodontitis’/exp OR periodontitis) AND (‘dental pulp stem cell’/exp OR ‘dental pulp stem cell’ OR ((‘dental’/exp OR dental) AND (‘pulp’/exp OR pulp) AND (‘stem’/exp OR stem) AND (‘cell’/exp OR cell))) AND (‘clinical’/exp OR clinical)) OR ((‘periodontal regeneration’/exp OR ‘periodontal regeneration’ OR (periodontal AND (‘regeneration’/exp OR regeneration)) OR ‘periodontitis’/exp OR periodontitis) AND (‘gingival mesenchymal stem cell’/exp OR ‘gingival mesenchymal stem cell’ OR (gingival AND mesenchymal AND (‘stem’/exp OR stem) AND (‘cell’/exp OR cell))) AND (‘clinical’/exp OR clinical)) OR ((‘periodontal regeneration’/exp OR ‘periodontal regeneration’ OR (periodontal AND (‘regeneration’/exp OR regeneration)) OR ‘periodontitis’/exp OR periodontitis) AND (‘umbilical cord mesenchymal stem cell’/exp OR ‘umbilical cord mesenchymal stem cell’ OR ((‘umbilical’/exp OR umbilical) AND cord AND mesenchymal AND (‘stem’/exp OR stem) AND (‘cell’/exp OR cell))) AND (‘clinical’/exp OR clinical)) OR ((‘periodontal regeneration’/exp OR ‘periodontal regeneration’ OR (periodontal AND (‘regeneration’/exp OR regeneration)) OR ‘periodontitis’/exp OR periodontitis) AND (‘bone marrow mesenchymal stem cell’/exp OR ‘bone marrow mesenchymal stem cell’ OR ((‘bone’/exp OR bone) AND (‘marrow’/exp OR marrow) AND mesenchymal AND (‘stem’/exp OR stem) AND (‘cell’/exp OR cell))) AND (‘clinical’/exp OR clinical))	1389
Scopus	TITLE-ABS-KEY ((( periodontal AND regeneration OR periodontitis) AND stem AND cell AND clinical) OR ((periodontal AND regeneration OR periodontitis) AND mesenchymal AND stem AND cell AND clinical) OR ((periodontal AND regeneration OR periodontitis) AND mesenchymal AND stromal AND cell AND clinical) OR ((periodontal AND regeneration OR periodontitis) AND dental AND stem AND cell AND clinical) OR ((periodontal AND regeneration OR periodontitis) AND periodontal AND ligament AND stem AND cell AND clinical) OR ((periodontal AND regeneration OR periodontitis) AND dental AND pulp AND stem AND cell AND clinical) OR ((periodontal AND regeneration OR periodontitis) AND gingival AND mesenchymal AND stem AND cell AND clinical) OR ((periodontal AND regeneration OR periodontitis) AND umbilical AND cord AND mesenchymal AND stem AND cell AND clinical) OR ((periodontal AND regeneration OR periodontitis) AND bone AND marrow AND mesenchymal AND stem AND cell AND clinical))	833
Wanfang	All:((periodontal regeneration OR periodontitis) AND stem cell AND clinical) OR All:((periodontal regeneration OR periodontitis) AND mesenchymal stem cell AND clinical) OR All:((periodontal regeneration OR periodontitis) AND mesenchymal stromal cell AND clinical) OR All:((periodontal regeneration OR periodontitis) AND dental stem cell AND clinical)) OR All:((periodontal regeneration OR periodontitis) AND umbilical cord mesenchymal stem cell AND clinical) OR All:((periodontal regeneration OR periodontitis) AND bone marrow mesenchymal stem cell AND clinical)	214
CNKI	(All Text%(periodontal regeneration OR periodontitis) AND stem cell AND clinical) OR (All Text%(periodontal regeneration OR periodontitis) AND mesenchymal stem cell AND clinical) OR (All Text%(periodontal regeneration OR periodontitis) AND mesenchymal stromal cell AND clinical) OR (All Text%(periodontal regeneration OR periodontitis) AND dental stem cell AND clinical) OR (All Text%(periodontal regeneration OR periodontitis) AND periodontal ligament stem cell AND clinical) OR (All Text%(periodontal regeneration OR periodontitis) AND dental pulp stem cell AND clinical) OR (All Text%(periodontal regeneration OR periodontitis) AND gingival mesenchymal stem cell AND clinical) OR (All Text%(periodontal regeneration OR periodontitis) AND umbilical cord mesenchymal stem cell AND clinical) OR (All Text%(periodontal regeneration OR periodontitis) AND bone marrow mesenchymal stem cell AND clinical)	2413

CENTRAL: Cochrane Central Register of Controlled trials; WOS: Web of Science; CNKI: China national knowledge infrastructure

### Data extraction and quality assessment

For each eligible study, data sheets were used to collect the extracted information independently by two investigators (L.S. and X.Y.D.). Disagreements were resolved through discussion or the judgment of other investigators (H.F.K., H.L.S. and C.Y.). The extracted information includes: characteristics of the study including clinical trial type, follow-up period, primary outcome, information of the patients including age and case number, characteristics of the clinical intervention including stem cell type or source, cell number, scaffold or free, the differences between stem cell-based therapy and cell free control group.

The risk of bias of the included studies was assessed with Cochrane Collaboration’s tool. The evaluation index contained seven items as random sequence generation, allocation concealment, blinding of participants and personnel, blinding of outcome assessment, incomplete outcome data, selective reporting and other bias. The above items are evaluated as “yes” (low risk of bias), “unclear” (unclear risk of bias) and “no” (high risk of bias).

### Data analysis and statistical methods

The extracted data were analyzed using Review Manager 5.4 software from Cochrane Library. For continuous data, the mean difference (MD) and 95% confidence interval (CI) were used to demonstrate efficacy analysis statistics, and P < 0.05 was considered statistically significant. The Cochrane Q-test and I^2^ statistics were used to analyze the amount of heterogeneity among the studies (P < 0.10 indicate significance, and I² > 50% indicate high heterogeneity). If there is no or little heterogeneity among the included studies (P ≥ 0.10, I^2^ ≤ 50%), and the fixed-effect model was used for analysis. When the included results showed significant heterogeneity (P < 0.10, I^2^ > 50%), the random effects model was used for analysis, and subgroup analysis were performed. In subgroup analysis, if the data in one or more subgroups exhibited significant heterogeneity (P < 0.10, I^2^ > 50%), the random effects statistical model were used for total data analysis.

### Certainty of evidence

Two independent investigators (L.S. and H.F.K.) assessed the certainty of evidence for each outcome using a grading of recommendations assessment, development, and evaluation (GRADE) system (GRADEpro GDT, https://gradepro.org/). The levels of certainty for outcomes based on five domains, including risk of bias, inconsistency, indirectness, imprecision, and publication bias. Discrepancies were resolved through consultation with other investigators (X.Y.D., H.L.S. and C.Y.).

## Results

### Search and selection of studies

**Fig. 1:**
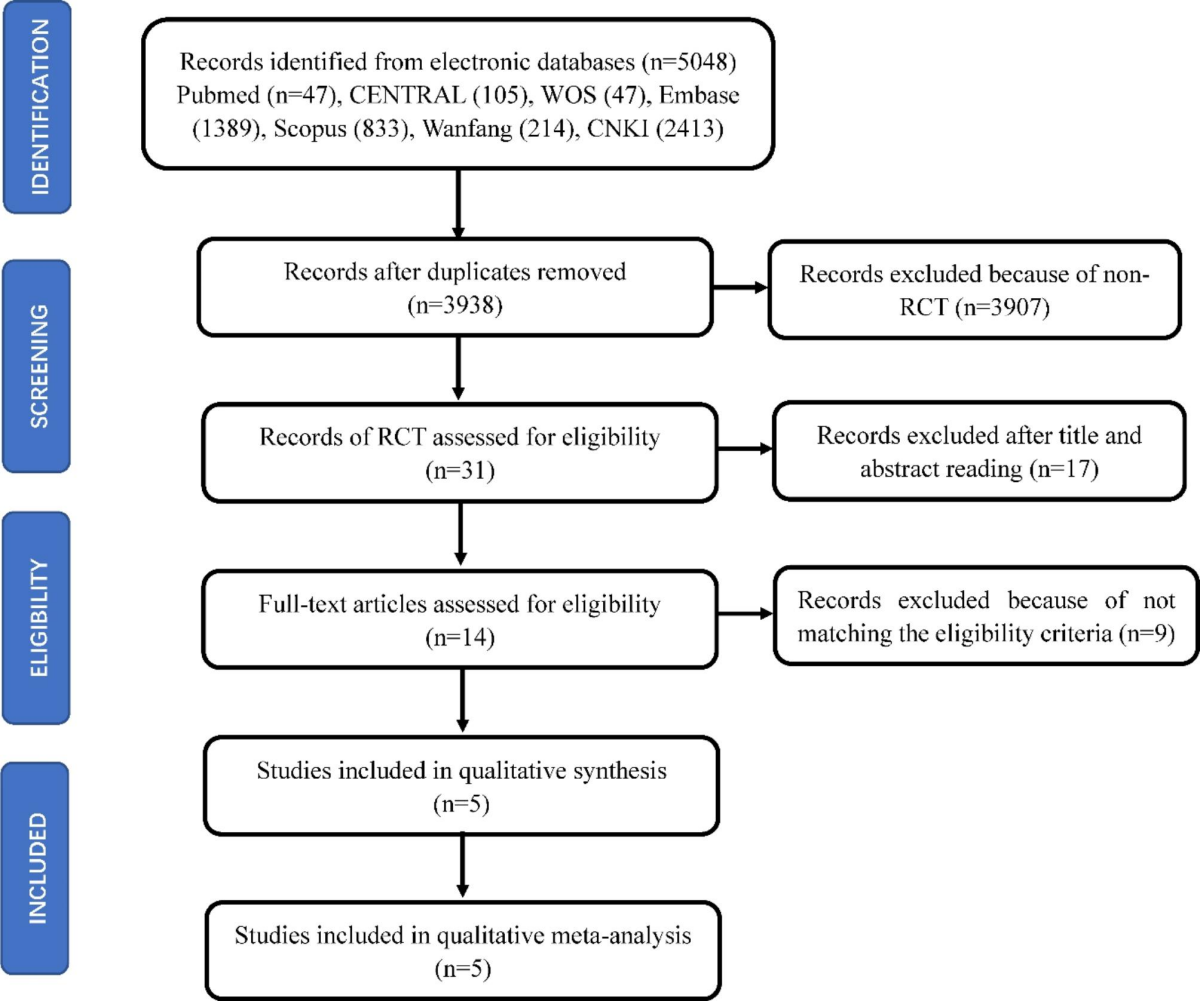
Flow diagram of the literature search for studies included in meta-analysis

After the articles were searched in PubMed, CENTRAL, WOS, Embase, Scoups, Wanfang and CNKI databases with the search strategy of Table 1, 5048 records were yielded during the preliminary searching. 1110 records were excluded because of duplication. 3907 records were excluded because of non-RCT. 17 records were removed after examination of the title and abstract found that they did not focus on stem cell-based therapies or periodontal regeneration, or were only in vitro studies. After the full texts had been assessed, 9 articles were excluded due to non-matching eligibility criteria. Finally, 5 eligible clinical studies related to stem cell-based therapy for periodontal regeneration were included in the qualitative and meta-analysis. The PRISMA diagram is presented in Fig. 1.

### Characteristics of the included studies

The characteristics of the 5 eligible studies are presented in Table 2. These studies were published from 2016 to 2021. 118 patients were enrolled in these studies, and the age ranged from 20 to 70. Autologous alveolar bone marrow mesenchymal stem cells (aABMMSCs, 1 study), autologous periodontal ligament stem cells (aPDLSCs, 2 studies), autologous dental pulp stem cells (aDPSCs, 1 study), autologous gingival fibroblasts and their associated mesenchymal stem cells (aGF-GSCs, 1 study) based therapy were included in this analysis. The follow-up period ranged from 3 weeks to 12 months. Some of the primary outcomes were not included in more than two studies, such as full-mouth plaque index, full-mouth plaque score, full-mouth bleeding score and so forth, thus these data were not included into analysis.

**Table 2:** Characteristics of the included studies

Author and year	Type	Cell type	Case number(TEST/CTRL)	Age	Clinical intervention		Follow-up period	Primary outcome
					Test	Control		
Apatzidou 2021	RCT	Autologous alveolar bone marrow mesenchymal stem cells (aABMMSCs)	19 patients(9/10)	20–68	A biocomplex comprising aABMMSCs, prepared under clinical-grade, current Good Manufacturing Practice(cGMP) conditions suspended into autologous fibrin/platelet lysate (aFPL), and loaded into a collagen fleece was transplanted into the osseous defect.	Collagen fleece enriched with aFPL devoid of stem cells filled the osseous defect or the defects were treated by the Minimal Access Flap	12 months	CAL; PPD; REC; CEJ-BD; BC-BD; WD.
Sánchez 2020	RCT	Autologous periodontal ligament-derived mesenchymal stem cells (aPDLSCs)	20 patients(9/10)^a^	25–70	The experimental treatment consisted of 1 × 10^7^ aPDLSCs incubated for 1 h (37ºC, 5% CO_2_ and 95% humidity) in 100 mg of a xenogeneic bone substitute.	The control treatment comprised the use of the same xenogeneic bone substitute seeded in 200 μm of physiological saline solution.	12 months	FMPI; CAL; PPD; REC.
Abdal-Wahab 2020	RCT	Autologous gingival fibroblasts and their associated mesenchymal stem cells (aGF-GSCs)	20 patients(10/10)	32–50	Ten intrabony periodontal defects received their cultured aGF-GSCs on a beta-tricalcium phosphate scaffold (β- TCP) followed non-perforated collagen membrane coverage	Ten intrabony periodontal defects received β-TCP followed by non-perforated collagen membrane.	6 months	CAL; VPD(PPD); RBG.
Ferrarotti 2018	RCT	Autologous dental pulp stem cells (aDPSCs)	29 patient(15/14)	39–69	The obtained micro-grafts enriched in aDPSCs were endorsed onto a collagen sponge scaffold. The collagen sponge was provided to the masked surgeon who filled the intrabony defect. The flaps were repositioned and tension-free primary flap closure was obtained using horizontal internal mattress and interrupted sutures	The collagen sponge was provided to the masked surgeon who filled the intrabony defect. The flaps were repositioned and tension-free primary flap closure was obtained using horizontal internal mattress and interrupted sutures	12 months	FMPS; FMBS; PPD; CAL; REC; IBD(BC-BD).
Chen 2016	RCT	Autologous periodontal ligament-derived mesenchymal stem cells (aABMMSCs)	30patients, 41 teeth (20/21)	18–65	A standard initial preparation, including oral hygiene instruction, full-mouth scaling, and root planning before surgical treatment. Bio-oss®/cell sheets were administered only to the bony defect region	A standard initial preparation, including oral hygiene instruction, full-mouth scaling, and root planning before surgical treatment. Bio-oss® only were administered only to the bony defect region.	12 months	BDP(CEJ-BD); CAL; PD; GR(REC).

CAL: clinical attachment levels; PPD: probing pocket depth; REC: recession of the gingival margin; CEJ-BD: linear distance from cementoenamel junction to bottom of defect; BC-BD: linear distance from bone crest to bottom of defect; WD: width of defect; FMPI: full-mouth plaque index; VPD: vertical pocket depth; RBG: Radiographic bone gain; FMPS: full-mouth plaque score; FMBS: full-mouth bleeding score; IBD: intrabony defect depth; BDP: the distance from the deepest part of the defect to the cementoenamel junction of the tooth; GR: gingival recession; ^a^One patient in the TEST group was lost to follow-up after 6 months.

### Risk of bias of the eligible studies

**Fig. 2:**
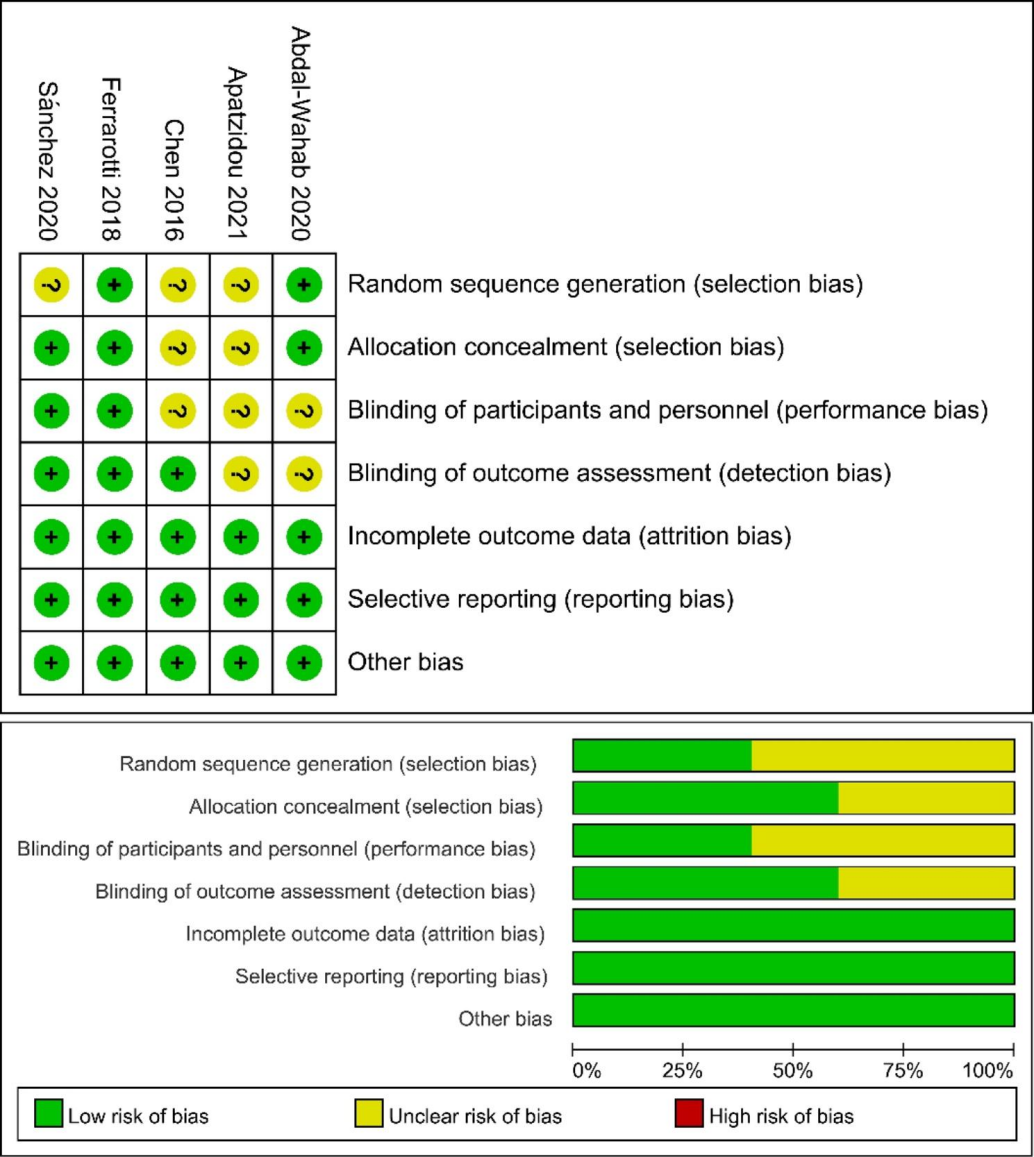
Quality assessment of the eligible studies. (**A**) Risk of bias graph: review authors’ judgements about each risk of bias item presented as percentages across all included studies. (**B**) Risk of bias summary: review authors’ judgements about each risk of bias item for each included study

The quality assessment of the eligible clinical studies is shown in Fig. 2. Three studies were evaluated as “unclear risk” of selection bias because of unclear description of random sequence generation. Two studies were evaluated as “unclear risk” of selection bias because of unclear description of allocation concealment. Three studies were evaluated as “unclear risk” of performance bias because of unclear description of blinding of participants and personnel. Two studies were evaluated as “unclear risk” of detection bias because of unclear description of blinding of outcome assessment. Finally, all studies were classified as low risk of attrition, reporting and other bias.

### Level of evidence

The level of evidence was determined as high (CAL), and most comparisons were evaluated as moderate, such as PPD, REC, CEJ-BD and BC-BD (Table 3). The main reasons for downgrading the certainty of the evidence were inconsistency and imprecision, due to the confidence interval overlapped less, the P value of the heterogeneity test was very small, the I^2^ was larger, and funnel graph asymmetry.

**Table 3:** Level of the evidence by the Grading of Recommendations Assessment, Development, and Evaluation (GRADE)

	Certainty assessment						Effect	
Outcomes	Number of studies (participants)	Risk of bias	Inconsistency	Indirectness	Imprecision	Other considerations	Absolute (95% CI)	Certainty
CAL	8 (195)	not serious	not serious	not serious	not serious	publication bias undetected	MD **1.18 lower** (1.55 lower to 0.8 lower)	⨁⨁⨁⨁ High
PPD	9 (236)	not serious	serious^a^	not serious	not serious	publication bias undetected	MD **0.75 lower** (1.35 lower to 0.14 lower)	⨁⨁⨁◯ Moderate
REC	8 (216)	not serious	not serious	not serious	serious^b^	publication bias undetected	MD **0.23 lower** (0.54 lower to 0.08 higher)	⨁⨁⨁◯ Moderate
CEJ-BD	5 (161)	not serious	not serious	not serious	serious^b^	publication bias undetected	MD **0.57 lower** (1.14 lower to 0.01 higher)	⨁⨁⨁◯ Moderate
BC-BD	4 (96)	not serious	not serious	not serious	serious^b^	publication bias undetected	MD **0.95 lower** (1.67 lower to 0.23 lower)	⨁⨁⨁◯ Moderate

CAL: clinical attachment levels; PPD: probing pocket depth; REC: recession of the gingival margin; CEJ-BD: linear distance from cementoenamel junction to bottom of defect; BC-BD: linear distance from bone crest to bottom of defect; ^a^the confidence interval overlapped less, the P value of the heterogeneity test was very small, and the I^2^ was larger; ^b^funnel graph asymmetry

### Outcomes of stem cell-based therapy for periodontitis

#### CAL

**Fig. 3:**
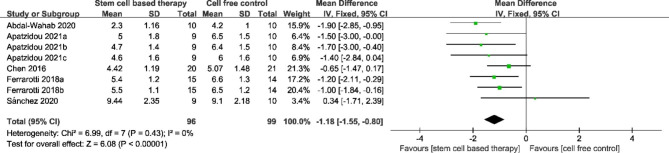
Forest plot for clinical attachment level. The analysis conducted to compare clinical attachment level between stem cell-based therapy and cell-free control therapy. A fixed-effects meta-analysis model (Mantel-Haenszel method) was used in this analysis

The primary outcome CAL between the stem cell-based group and the cell free control group was compared in five studies. The result of the Cochrane Q-test demonstrated great homogeneity (P = 0.43, I^2^ = 0%), and a fixed-effect model was conducted for analysis. Compared with cell free group, stem cell-based therapy group showed a significant decrease for CAL with a MD of − 1.18 mm (95% CI = − 1.55, − 0.80, P < 0.00001) from 3 to 12 months after surgery. (Fig. 3)

### PPD

**Fig. 4:**
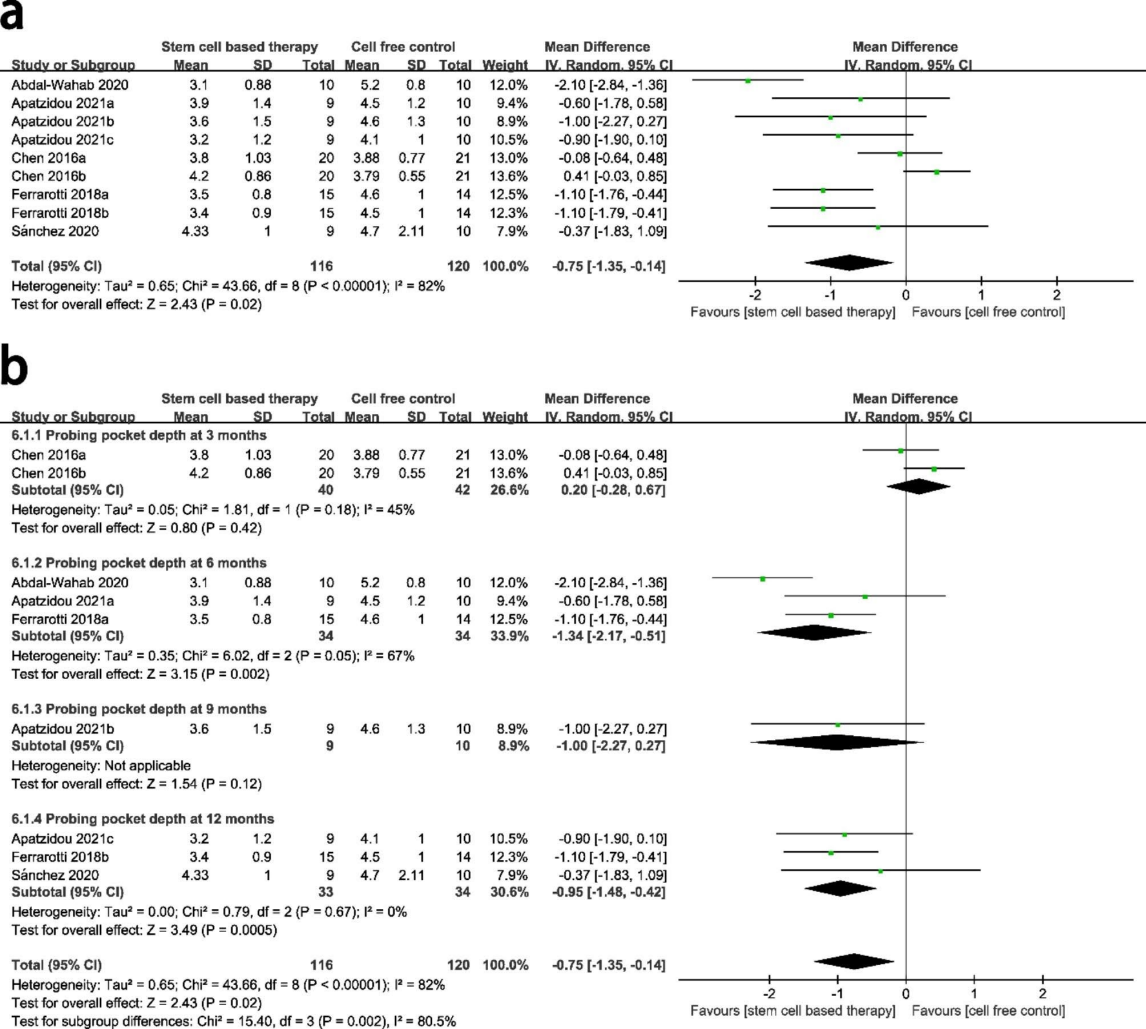
Forest plot for pocket probing depth. The analysis conducted to compare clinical attachment level pocket probing depth between stem cell-based therapy and cell-free control therapy. A random-effects meta-analysis model (Mantel-Haenszel method) was used in this analysis

A total of five studies compared PPD between the stem cell-based group and the cell free control group. There was high heterogeneity between the two groups (P < 0.00001, I^2^ = 82%), and a random-effect model was conducted for analysis. Compared with cell free group, stem cell-based therapy group showed a significant decrease for PPD with a MD of − 0.75 mm (95% CI = − 1.35, − 0.14, P = 0.02) from 3 to 12 months after surgery. (Fig. 4a)

Subgroup analysis showed a significant decrease for PPD at 6 and 12 months in the stem cell-based compared to the cell free control group (P = 0.002 and P = 0.0005 respectively), and there was no significant difference between the two groups at other follow-up periods. (Fig. 4b)

### REC

**Fig. 5:**
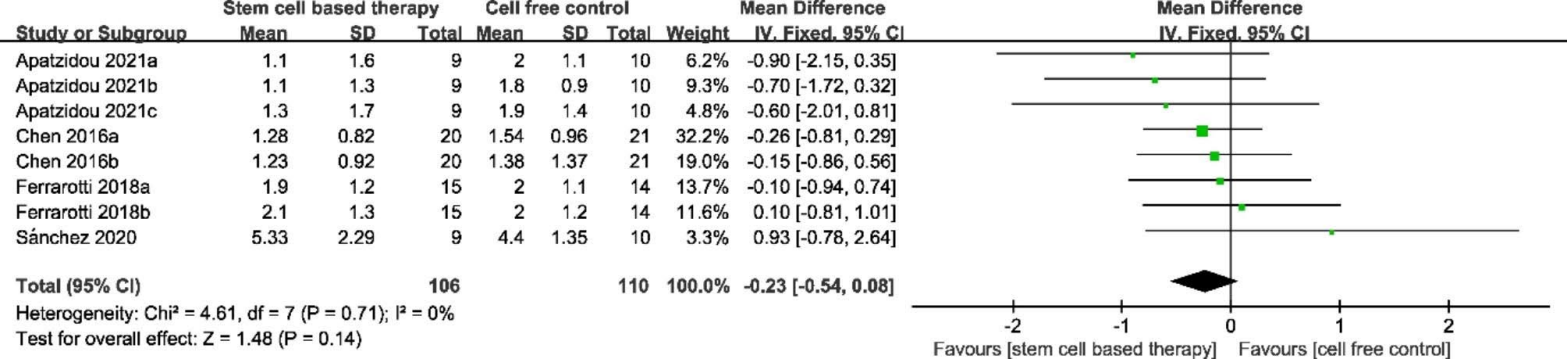
Forest plot for gingival recession. The analysis conducted to compare gingival recession between stem cell-based therapy and cell-free control therapy. A fixed-effects meta-analysis model (Mantel-Haenszel method) was used in this analysis

REC between the stem cell-based group and the cell free control group was compared in four studies. The result of merging showed great homogeneity (P = 0.71, I^2^ = 0%). The meta-analysis conducted using a fixed-effect model revealed a significant difference in REC between the two groups (MD = − 0.23, 95% CI = − 0.54, 0.08, P = 0.14) from 3 to 12 months after surgery. (Fig. 5)

### CEJ-BD

**Fig. 6:**
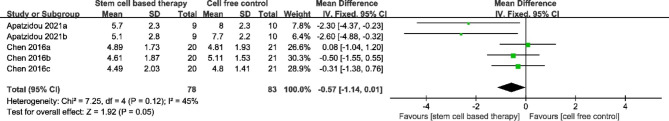
Forest plot for linear distance from cementoenamel junction to bottom of defect on radiographs at 12 months. The meta-analysis conducted to compare cementoenamel junction to bottom of defect between stem cell-based therapy and cell-free control therapy. A random-effects meta-analysis model (Mantel-Haenszel method) was used in this analysis

A total of two studies compared CEJ-BD between the stem cell-based group and the cell free control group. The meta-analysis conducted using a random-effect model for did not show significant difference in CEJ-BD between the two groups (MD = -0.57, 95% CI = − 1.14, 0.01, P = 0.05, I^2^ = 45). (Fig. 6)

### BC-BD

**Fig. 7:**
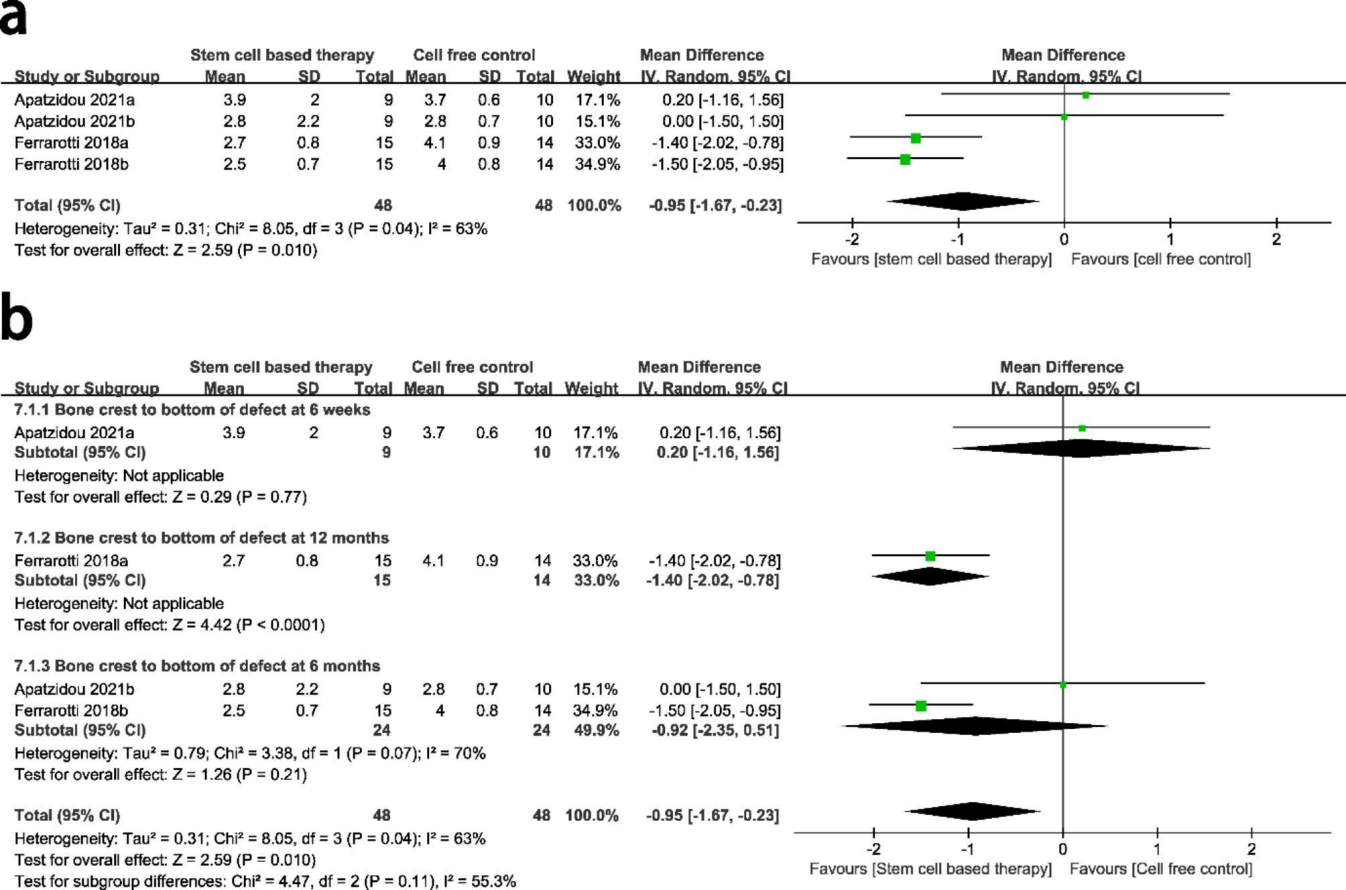
Forest plot for linear distance from bone crest to bottom of defect at 12 months. The meta-analysis conducted to compare bone crest to bottom of defect between stem cell-based therapy and cell-free control therapy. A random-effects meta-analysis model (Mantel-Haenszel method) was used in this analysis

A total of two studies compared BC-BD between the stem cell-based group and the cell free control group. There was high heterogeneity between the two groups (P = 0.04, I^2^ = 63%), and a random-effect model was conducted for analysis. Compared with cell free group, stem cell-based therapy group showed a significant improvement for BC-BD with a MD of − 0.95 mm (95% CI = − 1.67, − 0.23, P = 0.010) from 3 weeks to 12 months after surgery. (Fig. 7a)

Subgroup analysis showed a significant decrease for PPD at 6 months in the stem cell-based compared to the cell free control group (P < 0.0001), and there was no significant difference between the two groups at 3 weeks and 12 months. (Fig. 7b)

## Discussion

### Summary of evidence

Periodontitis as the most common cause of tooth loss in adults affects lots of people all over the world. The therapeutic cost of periodontal diseases is huge and are likely to increase along with the rapidly aging population [15]. Many studies have revealed that periodontal diseases can influence the risk of systemic conditions, including metabolic disease, cardiovascular disease, respiratory disease, neurodegenerative disease and even cancers [16–21]. However, previous clinical treatments can just control infections and arrest the disease progression, but cannot achieve complete periodontal regeneration. The development of stem cell research and regenerative medicine enables the novel cell-based therapeutics with better outcomes.

To date, mesenchymal stem cell can be isolated from many tissue sources. For the treatment of periodontal disease, dental stem cells (DSCs) seem to be the best candidate [22, 23]. Besides treating oral diseases, DSCs also exhibited great potential to restore neurological function after nerve injury because they originate from the cranial neural crest [24–26]. Previous studies have demonstrated that five main DSCs were isolated from dental pulp of permanent teeth, dental pulp of exfoliated deciduous teeth, apical papilla, periodontal ligament and dental follicle tissues [9] Later in 2009, GSCs were firstly isolated from gingival tissue [27]. In the past decades, these DSCs have been used in tissue engineering and disease treatments, such as oral, neurologic, diabetic and auto-immune conditions, etc. Thus, the stem cell researchers suggested cryopreservation and banking of DSCs, which will provide a desirable approach for stem cell-based therapy and tissue engineering [28, 29].

For the outcomes of stem cell-based therapy in periodontal disease of preclinical studies, previous meta-analysis demonstrated that stem cells have a favorable effect on new bone, cementum and PDL formation in periodontal defects after analyzing the data of 39 preclinical studies [30]. Another meta-analysis also showed that PDLSCs and bone marrow mesenchymal stem cells have consistently exhibited therapeutic benefits on newly formed bone, newly formed cementum and newly formed periodontal ligament in preclinical studies of periodontal defect animal models. Meanwhile, PDLSCs are more superior for newly formed bone and newly formed cementum to GSCs [31].

As mentioned above, it has been proven that stem cell transplantation enhanced the periodontal tissues regeneration in various animal models. Therefore, stem cell researchers appeal that it is time to move from preclinical studies to human clinical trials [32]. Could stem cell transplantation achieve similar effects in human patients?

A previous meta-analysis of clinical application of mesenchymal stem cells in periodontal regeneration showed that there was a small but significant difference between stem cell and cell free control groups was found for CAL at three months, but not for PPD and REC [3]. Actually, there was a mistake in this meta-analysis: one of the RCTs included in the analysis did not contain data of CAL, PPD and REC at three months but at six months [6]. Another meta-analysis about the application of stem cells for the regeneration of periodontal defects has been published recently, which demonstrated that there was a significant difference between experimental and control groups was found for CAL, PPD, REC and bone defect depth [33]. For most of the included RCTs in that meta-analysis, the difference between stem cell group and cell-free group was whether or not cell added. However, that meta-analysis included an RCT, which contained a control group was treated by an open flap debridement alone, but the test group treated with combination of stem cells cultured on beta tricalcium phosphate with recombinant human platelet derived growth factor-BB [34]. The differences of the therapeutic results in this RCT were thus based on the combination of stem cell, scaffold and growth factor, but not stem cell only, which might exaggerate the role of stem cells. Therefore, this RCTs should not be included in the meta-analysis which focused on the therapeutic effect of stem cells. As the number of RCTs increases, we newly assessed the current clinical efficacy of stem cell transplantation for patients with periodontitis. Three typical types of autologous DSCs (aDPSCs, aPDLSCs, aGF-GSCs) and aABMMSCs were used in these five eligible studies for the periodontal regeneration. Compared with cell-free treatments, the analysis results demonstrated that stem cell-based therapy could decrease the level of CAL, PPD and BC-BD at follow-up periods; however, it presented insignificant effects on REC and CEJ-BD.

Compared with the results of preclinical treatments, the clinical results included in this meta-analysis may not have a satisfactory outcome in REC and CEJ-BD. In contrast to the number of preclinical studies, there are not enough high quality RCTs to analyze at present. Meanwhile, the reasons for the discrepancy between preclinical and clinical studies should be carefully considered. For instance, it is unknown whether animal models can simulate the pathogenesis of periodontal disease, and whether the extent of periodontal damage in animal models is comparable to that in clinical moderate to severe periodontitis. Besides, exploring the appropriate stem cells to solve clinical problems, as well as to obtain better clinical outcomes should also be considered seriously. Although some previous studies demonstrated that PDLSCs might be the first choice for periodontal regeneration [3, 35], dental follicle give rise to periodontal tissues, including cementum, alveolar bone and periodontal ligament [36], and other comparative studies also revealed that DFSCs exhibited a stronger capacity for regeneration of cementum and periodontal attachment than PDLSCs [37, 38]. Therefore, DFSCs can be considered as a better candidate cell source for future clinical applications [39].

### Strengths and limitations

Although strict inclusion and exclusion criteria, and strict quality evaluation were enforced in this meta-analysis, the following limitations still existed. First, the inclusion criteria in this meta-analysis mainly require the studies were clinical studies and concern stem cell-based therapy, therefore only five RCTs were enrolled in the qualitative and meta-analysis. Second, the stem cells and scaffold materials used in the RCTs were not unified. For example, aABMMSCs were used in 1 study; aPDLSCs were used in 2 studies; aDPSCs were used in 1 study and aGF-GSCs were used in 1 study. The scaffold materials were collagen fleece, collagen sponge, xenogeneic bone substitute, Bio-Oss natural bone substitute and beta-tricalcium phosphate. Third, some of the RCTs included in this meta-analysis with small sample size, single-center characteristics, non-uniform outcome measures and different follow-up time, which make part of the data cannot be analyzed. Fourth, some of the RCTs present uncertain risk of selection bias due to lack of the clear description of random sequence generation, allocation concealment, blinding of participants and personnel and blinding of outcome assessment. Thus, the results of the meta-analysis should be evaluated scientifically and cautiously. The high-quality RCTs with large sample size, multi-blind, multicentric are still indeed required. Moreover, the methodological and normative clinical study protocol, including inclusion criteria, cell type, outcome measures, follow-up time etc. should be established and executed by the future clinical researchers.

## Conclusion

In conclusion, compared with the cell-free group, the results of this meta-analysis demonstrated that stem cell-based therapy showed better therapeutic effects on CAL, PPD and BC-BD at follow-up periods, but there’s no significant effects on REC and CEJ-BD. However, in consideration of the limitations of the included studies, the strength of the results in this analysis is affected to a certain extent. Therefore, large samples, multicenter, multi-blind, methodological and normative RCTs are required and expected to further validate the therapeutic effect of stem cell-based therapy on moderate-severe periodontitis, and provide reliable evidences of evidence-based medicine for its clinical application.

## Data Availability

All data generated or analyzed during this study are available from the corresponding author.

## Abbreviations

RCTs: Randomized controlled trials
CAL: Clinical attachment level
PPD: Pocket probing depth
MSCs: Mesenchymal stem cells
SHED: Stem cells from human exfoliated deciduous teeth
DFSC: Dental follicle stem cells
SCAP: Stem cells from apical papilla
DPSCs: Dental pulp stem cells
PDLSCs: Periodontal ligament stem cells
GSCs: Gingival stem cells
REC: Gingival recession
CEJ-BD: Linear distance from cementoenamel junction to bottom of defect on radiographs
BC-BD: Linear distance from bone crest to bottom of defect
CENTRAL: Cochrane Central Register of Controlled trials
aABMMSCs: Autologous alveolar bone marrow mesenchymal stem cells
aPDLSCs: Autologous periodontal ligament stem cells
aDPSCs: Autologous dental pulp stem cells
aGF-GSCs: Autologous gingival fibroblasts and their associated mesenchymal stem cells
MD: Mean difference
